# Endothelin-1 Production by Normal Human Cultured Keratinocytes and its Regulation

**DOI:** 10.1155/S096293519400061X

**Published:** 1994

**Authors:** Hajime Inoue, Nagaoki Wakisaka, Nobuhiro Tane, Kazumasa Ando, Emiko Isono, Miwa Yamanaka, Masaki Aihara, Hirotomo Ishida

**Affiliations:** Department of Plastic and Reconstructive Surgery St Marianna University School of Medicine 2-16-1 Sugao Miyamae Kawasaki 216 Japan

## Abstract

The possibility that cultured keratinocytes produce endothelins were investigated. The results showed that cultured keratinocytes derived from normal human skin produce endothelin-1. Moreover, keratinocyte endothelin-1 production was completely inhibited by the presence of actinomycin D in the medium. As in the case of endothelial cells, recombinant interleukin-1β was capable of promoting endothelin-1 production in keratinocytes, whereas herapin inhibited it. Thrombin also inhibited endothelin-1 production. These results indicate that the mechanism of endothelin-1 production in keratinocytes is slightly different from the mechanism in vascular endothelial cells.


Mediators of Inflammation 3, 433–437 (1994)

### Endothelin-1 production by normal human cultured keratinocytes and its regulation

Hajime Inoue,[CA] Nagaoki Wakisaka, Nobuhiro Tane, Kazumasa Ando, Emiko Isono, Miwa Yamanaka, Masaki Aihara and Hirotomo Ishida

Department of Plastic and Reconstructive Surgery, St Marianna University School of Medicine, 2-16-1 Sugao, Miyamae, Kawasaki 216, Japan

[CA] Corresponding Author

**The possibility that cultured keratinocytes produce endothelins were investigated. The results showed that cultured keratinocytes derived from normal human skin produce endothelin-1. Moreover, keratinocyte endothelin-1 production was completely inhibited by the presence of actinomycin D in the medium. As in the case of endothelial cells, recombinant interleukin-1β was capable of promoting endothelin-1 production in keratinocytes, whereas herapin inhibited it. Thrombin also inhibited endothelin-1 production. These results indicate that the mechanism of endothelin-1 production in keratinocytes is slightly different from the mechanism in vascular endothelial cells.**

**Key words:** Endothelin-1, Heparin, Interleukin-1, Keratinocytes, Thrombin

### Introduction

Endothelin is a peptide that causes potent contraction of vascular smooth muscle.[1] Endothelin not only has a potent constricting action on smooth muscle cells, but appears to promote arachidonic acid metabolism and the production of endothelium-derived relaxing factor (EDRF).[2] Moreover, its promotion of fibroblast proliferation has been confirmed *in vitro*.[3]

Although endothelin was previously thought to be a specific product of vascular endothelial cells. It has since been found to be produced by tracheal epithelial cells and nerve cells in the central nervous system, where it acts as a neurotransmitter.[4,5] Recently, the authors proposed that endothelin-1 may participate as an inflammatory mediator because endothelin-like immunoreactants or endothelin-1 appeared and increased at the wound surface after thermal injury.[6] Thus endothelin-1 may be capable of regulating various cell functions, however, it is still not clear whether keratinocytes produce endothelin-1.

In the present study, the authors investigated endothelin-1 production by cultured human keratinocytes and the mechanisms regulating it.

### Materials and Methods

*Keratinocyte cultures*: Excess skin obtained during plastic surgery (in particular skin grafting) was made available to culture normal human keratinocytes. The skin was finely cut (4 × 4 mm) and after being disinfected in 10% providone–iodine solution (Meiji Seika Co., Tokyo, Japan) for 2 min, it was exposed to phosphate-buffered saline (PBS, Dainihon Seiyaku Co., Tokyo, Japan) at ambient temperature for 10 min containing 0.02% disodium ethylene diamine tetraacetate (EDTA; Dojin Chemical Co., Tokyo, Japan). The minced skin was then digested in Dulbecco's modified Eagle medium (DME; Gibco BRL Co., Grand Island, NY) containing 0.25% trypsin (Gibco BRL) for 18 h at 4°C, and washed twice with PBS. The enzyme-treated skin was stirred for 1 h in DME medium containing 20% foetal calf serum (Gibco BRL), antibiotics (penicillin G, kanamycin (Meiji Seika) and amphotericin B (Gibco BRL)), was filtered through a Cell strainer (Becton Dickinson Japan Co., Tokyo, Japan), and the skin residue was removed. Thus keratinocytes were obtained from skin as free cells. The free cell suspension was then centrifuged at 1200 r.p.m. for 5 min at 4°C. Keratinocyte growth medium (KGM, modified MCDB 153 containing EGF, insulin, hydrocortisone and bovine pituitary extracts, Kurasikiboseki Co., Osaka, Japan) was added to the cell pellet and mixed to obtain a homogenous suspension. The keratinocytes were cultured by the Boyce method,[7] and the medium was exchanged after an initial 24 h of culturing and every 2 days thereafter until confluence. When cultured primary keratinocytes had reached confluence, they were re-suspended by adding PBS containing 0.1% trypsin and EDTA, then passaged by the same procedure.

*Endothelin-1 production experiments*: When cultured keratinocytes became confluent in the second passage, they were re-suspended and seeded at a density of 5000 cells/well (2500 cells/$cm^2$) on to 24-

© 1994 Rapid Communications of Oxford Ltd well culture dishes. The cells were cultured at 37°C under conditions of 5% $CO_2$/air and 100% humidity. One ml of KGM with several concentrations of drugs (without drugs and with rIL-1β (Wako Pure Chemical Co., Osaka, Japan): 0.1, 1.0 and 5.0 U/ml, thrombin (Sigma Chemical Co., St Louis, MO); 0.2, 2.0 and 20.0 U/ml, heparin (Sigma); 1.0 and 10.0 U/ml) was added to similarly cultured cells according to the experimental schedule shown in Table 1 after they had reached confluence, and the mixture was incubated at 37°C. At the same time, a culture containing 40 nM of actinomycin D (Sigma) in the KGM in addition to the above drugs, was prepared. The medium obtained after 24 h of incubation reaction was centrifuged at 1 500 r.p.m. for 10 min at 4°C and stored at –80°C until endothelin-1 was measured.

*Measurements of endothelin-1*: Endothelin-1 was measured with an endothelin-1 EIA kit purchased from IBL Co. (Gunma, Japan). The procedure was as follows. A 96-well microplate pre-coated with monoclonal endothelin-1 antibody was washed twice with 0.2 ml of PBS containing 0.05% Tween 20 (wash buffer). The medium as specimens, or various concentrations of authentic endothelin-1 for calibration, were added, 0.1 ml per well of the pre-conditioned microplate. The plate was incubated for 18 h at 4°C, and then all of the wells were rinsed six times with 0.2 ml of wash buffer. The following 0.1 ml of the 3 μg/ml peroxidase-conjugated polyclonal endothelin-1 antibody solution was added to each well of the microplate. After the plate was again incubated for 30 min at 37°C, each well was washed six times following the same procedure. Then the 0.1 ml of enzymatic substrates for peroxidase (0.1 M potassium phosphate buffer, pH 7.0, containing 0.4 mg/ml *o*-phenylenediamine and 0.3% hydrogen peroxide) was added to each of the wells of the reacted plate. After incubating for 15 min at ambient temperature, the reaction was stopped by adding 0.1 ml of 1 N sulphuric acid. The optimal density of each well at 492 nm was determined using a plate reader (Bio-Rad Japan Co., Tokyo, Japan). The endothelin-1 content of the medium was calculated by comparison with the calibration curve of authentic endothelin-1. A PC-9801 type DA on-line data processor (NEC Co., Tokyo, Japan) was used to make the calculations. This EIA method has good reliability to 4.8 pg/ml (0.48 pg/well) on the calibration curve for endothelin-1, but the on-line data processor calculates to less than 4.8 pg/ml. The values less than 4.8 pg/ml were noted as <4.8 pg/ml, even though the on-line data processor calculates the values less than 4.8 pg/ml. Moreover, the experiments were designed to examine the effect of serum, thrombin, heparin and rIL-1β on this EIA system. As a consequence, these biological active substances, or until at least a 20% concentration of serum, did not affect use in this EIA assay.

*Statistical analysis*: The statistical significance of the results was evaluated using Student's *t*-test. Values are expressed as means ± SEM.

### Results

*Endothelin-1 production by keratinocytes*: Cultured human keratinocytes produced endothelin-1. At 24 h after the start of culture, the endothelin-1 level in the medium was 23.79 ± 0.29 pg/well ($n = 4$). Endothelin-1 production by keratinocytes was completely inhibited by actinomycin D and could not be detected in the medium after being cultured for 24 h (Fig. 1).

*Regulation of endothelin-1 production in keratinocytes*: Production of endothelin-1 by cultured human keratinocytes in the presence of rIL-1β increased dose-dependently. As shown in Fig. 2, the endothelin-1 levels in the medium were 33.52 ± 1.62, 41.65 ± 3.58 and 56.84 ± 15.40 pg/well at rIL-1β concentrations of 0.1, 1.0 and 5.0 U/ml, respectively, compared with 23.79 ± 0.29 pg/well obtained

**Table 1**. Experimental schedule

Confluent keratinocytes
↓
The various drugs listed below were added in the KGM

| | Control | rIL-1β | Thrombin | Heparin |
|---|---|---|---|---|
| With AMD[a] | only medium | 0.1 U/ml | 0.2 U/ml | not done |
| | | 1.0 U/ml | 2.0 U/ml | 1.0 U/ml |
| | | 5.0 U/ml | 20.0 U/ml | 10.0 U/ml |
| without AMD | only medium | 0.1 U/ml | 0.2 U/ml | not done |
| | | 1.0 U/ml | 2.0 U/ml | 1.0 U/ml |
| | | 5.0 U/ml | 20.0 U/ml | 10.0 U/ml |

↓
The keratinocytes were incubated for 24 h at 37°C
↓
The medium was collected as a specimen for assay

[a]AMD: 40 nM actinomycin D.

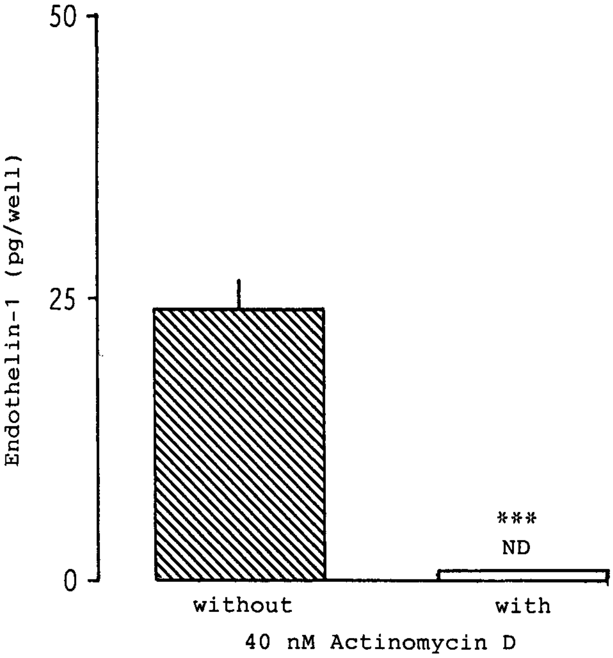

FIG. 1. Production of endothelin-1 (ET-1) from cultured human keratinocytes. The keratinocytes were incubated for 24 h at 37°C under 5% $CO_2$/air condition. Each column represents mean ± SEM ND: not detectable. ***$p < 0.001$: compared with the value obtained without actinomycin D.

without rIL-1β ($n$ = 4). The response in the presence of thrombin, however, was different. At low concentrations, thrombin decreased endothelin-1 production, whereas at high concentrations approximately the same level of endothelin-1 were produced as when no thrombin was added, i.e. the levels were 7.74 ± 0.50, 14.33 ± 3.80 and 27.86 ± 2.54 pg/well respectively ($n$=4), as shown in Fig 3. The endothelin-1 content of the medium 24 h after culture without and with 1.0 and 10.0 U/ml heparin was 23.79 ± 0.29, 12.83 ± 7.20 and 12.00 ± 1.46 pg/well respectively ($n$ = 4), as shown in Fig. 4. The addition of heparin resulted in dose-dependent inhibition of endothelin-1 production by keratinocytes. The presence of actinomycin D, on the other hand, inhibited endothelin-1 production in all cases ($n$ = 4, Figs 1–4).

### Discussion

Endothelin-1 was discovered in 1988 and was initially considered to be a hypertensive peptide produced only by vascular endothelial cells.[1] Later, tracheal epithelial cells, renal tubules and mesangial cells were also found to be capable of producing endothelin-1.[2,8,9] Thus, in addition to affecting smooth muscles, endothelin-1 probably has wide-ranging effects on cells and influences organ function as a whole.

In the present study the authors used cultured keratinocytes in an attempt to show that normal human keratinocytes produce endothelin-1. The results showed that cultured keratinocytes spontaneously produce endothelin-1. Moreover, this

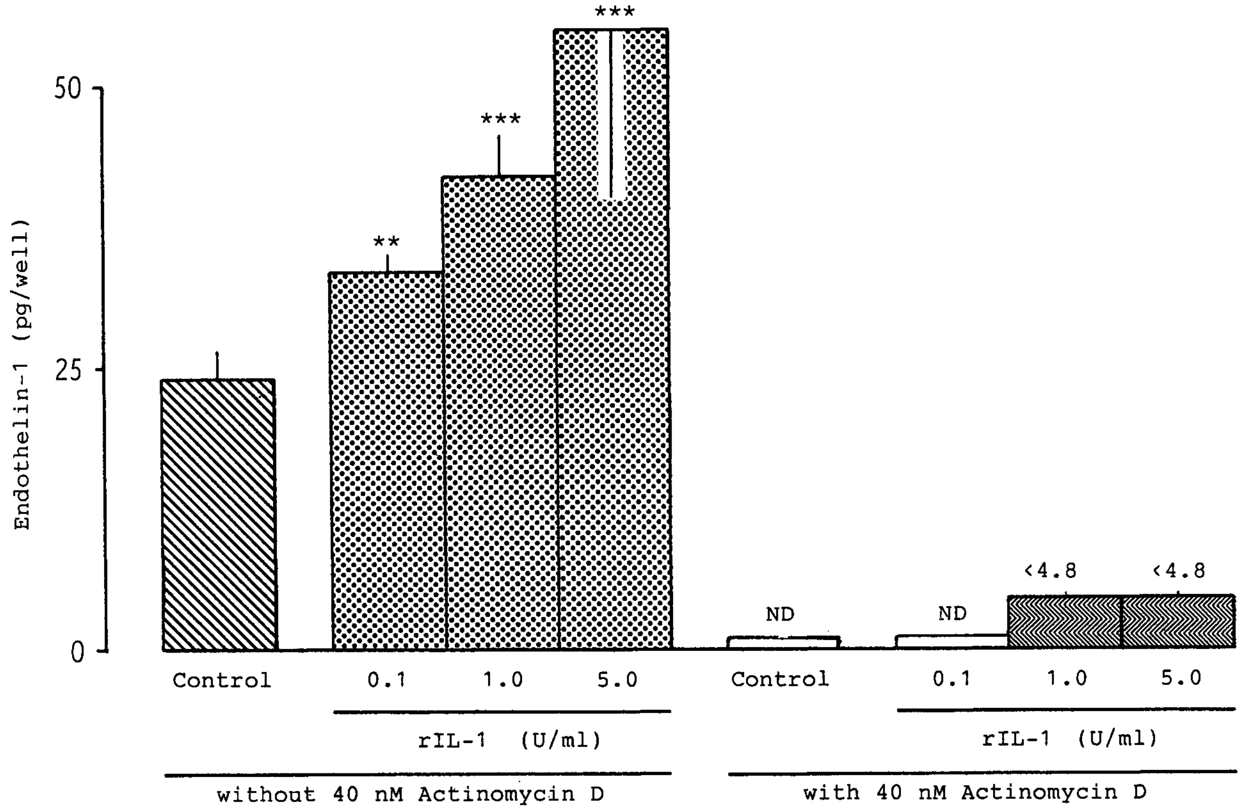

FIG. 2. Influence of recombinant interleukin 1β (rIL-1β) on endothelin-1 (ET-1) production of cultured human keratinocytes. The keratinocytes were incubated for 24 h at 37°C under 5% $CO_2$/air condition. Each column represents mean ± SEM. ND: not detectable. <4.8: trace level less than 4.8 pg/ml of minimum calibration range. **$p < 0.01$ and ***$p < 0.001$: compared with the value obtained by control (without rIL-1b).

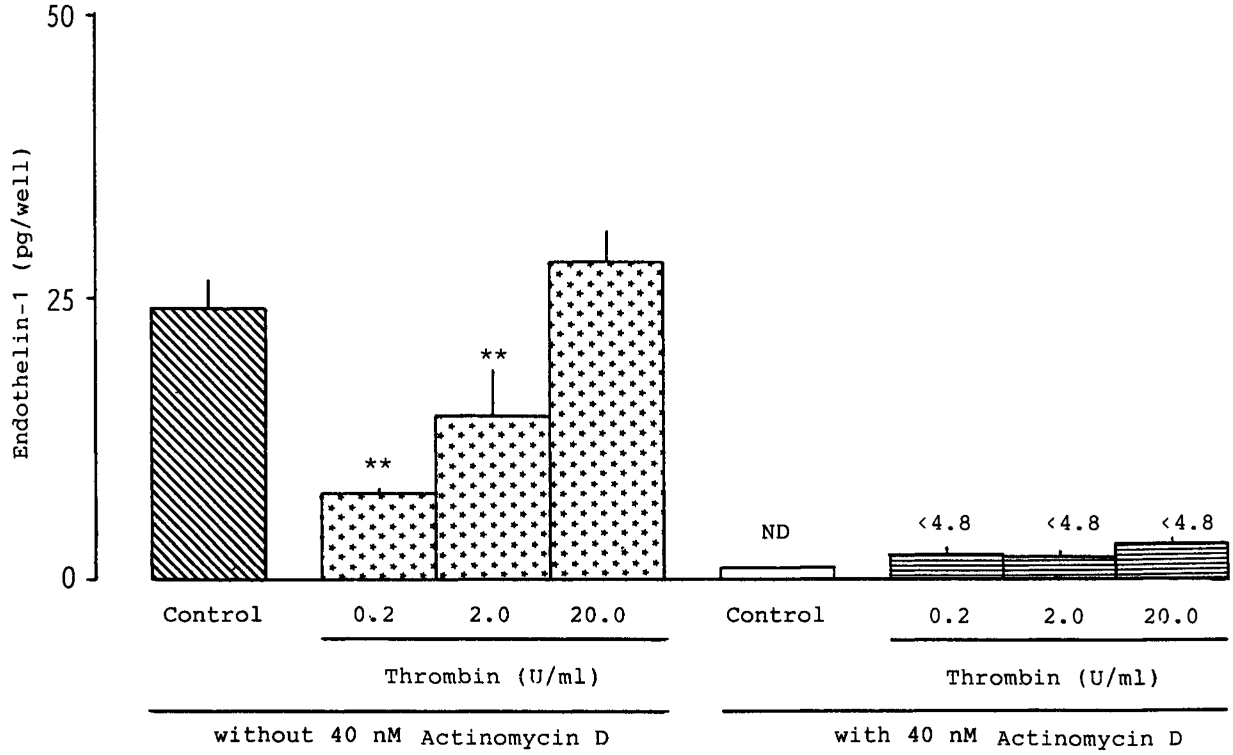

FIG. 3. Influence of thrombin on endothelin-1 (ET-1) production of cultured human keratinocytes. The keratinocytes were incubated for 24 h at 37°C under 5% $CO_2$/air condition. Each column represents mean ± SEM. ND: not detectable. 4.8: trace level less than 4.8 pg/ml of minimum calibration range. $^{**}p < 0.01$ and $^{***}p < 0.001$: compared with the value obtained by control (without thrombin).

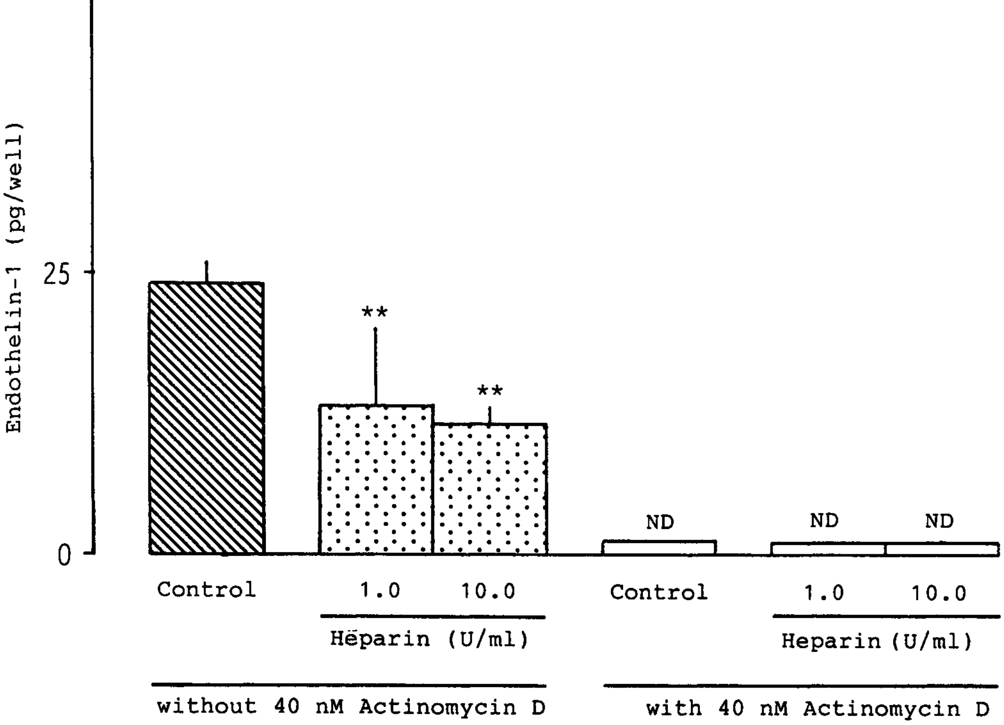

FIG. 4. Influence of heparin on endothelin-1 (ET-1) production of cultured human keratinocytes. The keratinocytes were incubated for 24 h at 37°C under 5% $CO_2$/air condition. Each column represents mean ± SEM. ND: not detectable. $^{**}p < 0.01$ and $^{***}p < 0.001$: compared with the value obtained by control (without heparin).

endothelin-1 production was completely inhibited by the addition of actinomycin D. This suggests that endothelin-1, as in the case of vascular endothelial cells, is not stored within the cells.

In 1991, Bull *et al.*[10] failed to detect the endothelin-1 production by keratinocytes using the indirect immunofluorescence technique. Yohn *et al.*,[11] however, demonstrated that cultured human keratinocytes could synthesize endothelin-1 at a rate of about 22 pg/1 × $10^6$ cells spontaneously at 24 h. The results we obtained were also about 24 pg/dish at 24 h in the same culture conditions. We therefore investigated the regulation of endothelin-1 synthesis by keratinocytes on the various bioactive substances.

Cytokines, such as IL-1 and tumour necrosis factor (TNF), activate endothelial C-kinase or calmodulin, and promote endothelin-1 gene expression and endothelin-1 secretion.[12,13] Thrombin enhances endothelial cell endothelin secretion via inositol metabolism in a similar manner.[14] The results of this study show that IL-1-mediated endothelin-1 production is identical to the response obtained from endothelial cells. Thus, IL-1-induced endothelin-1 production by keratinocytes may appear to be regulated by the same mechanism found in endothelial cells. However, the similarity of mechanisms should be confirmed by the experiment designed to compare the effects of various inhibitors of signal transduction pathway for IL-1 according to the detail reports of Katabami *et al.*[13] including expression of endothelin converting enzyme or big-endothelin-1 by cultured keratinocytes.

Keratinocytes are a plentiful source of the IL-1.[15] Moreover, it is known that IL-1 regulates keratinocytes function as an autocrine and/or paracrine mediator.[16] IL-1 release by keratinocytes can also control the stimulation of prostaglandin synthesis in arachidonic acid metabolism.[17] Based on these findings endothelin-1 production may also be regulated by IL-1 synthesis in keratinocytes. Thrombin reactivity, however, was different, and low thrombin concentrations (0.2–2.0 U/ml) decreased epithelial endothelin-1 production. In simultaneous comparative studies of vascular endothelial and tracheal epithelial cells, thrombin increased endothelin-1 production in a dose-dependent manner (data not shown). Accordingly, these results suggest that the inhibition mechanism of thrombin mediated endothelin-1 production in keratinocytes and endothelial cell may be different. Heparin, on the other hand, inhibited keratinocyte endothelin-1 production. This inhibition was attributed to heparin-induced inhibition of inositol metabolism and $Ca^{2+}$ influx, similar to that observed in endothelial cells.[18]

The above results demonstrate that bioactive substances can control the endothelin-1 synthesis by keratinocytes. In particular, IL-1 can stimulate endothelin-1 production by keratinocytes, but at present the role of the endothelin-1 produced is unclear. In recent studies Yada *et al.*[19] found that melanocytes contain large quantities of endothelin-1 receptor and that endothelin-1 could stimulate the cell proliferation and tyrosinase activity. Yohn *et al.*[11] concluded from the results of their investigation that endothelin-1 production by keratinocytes may participate in melanocyte proliferation and pigmentation. The increase in IL-1 mediated endothelin-1 synthesis by keratinocytes suggested the following possible sequence of events: (1) IL-1 production by keratinocytes increases in presence to UV radiation, etc.;[19] (2) this IL-1 stimulates endothelin-1 production by keratinocytes; and (3) the endothelin-1 promotes the proliferation or activation of tyrosinase in melanocytes; and finally (4) these processes may be a factor in the development of pigmentation *in vivo*. However, they also indicated that the mechanism of endothelin-1 production and secretion differs slightly from that observed in vascular endothelial cells. Future detailed studies on the influence of cell differentiation and skin inflammation on endothelin-1 production, including the problems raised by this study, are required.

### References

1. Yanagisawa M, Kurihara S, Kimura Y, Tomobe Y, Kobayashi M, Mitsui Y, Yazaki Y, Goto K, Masaki T. A novel potent vasoconstrictor peptide produced by bascular endothelial cells. *Nature* 1988; **32**: 411–415.
2. Warner TD, de Nucci T, Vane JR. Rat endothelin is a vasodilator in the isolated perfused mesentary of the rat. *Eur J Pharmacol* 1989; **159**: 325–326.
3. Takuwa N, Takuwa Y, Yanagisawa M, Yamashita K, Masaki T. A novel vasoactive peptide endothelin stimulates mitogenesis through inositol lipid turnover in Swiss 3T3 fibroblasts. *J Biol Chem* 1989; **264**: 7856–7861.
4. Nomura A, Uchida Y, Ishi Y, Kamezawa M, Ninomiya H, Saotome M, Ohse H, Hasegawa S. Endothelin, a bronchoconstrictor, is located in rat bronchial epithelial cells. *Am Rev Respir Dis* 1990; **141**: A290.
5. Yoshizawa T, Shinmi T, Giaid A, Yanagisawa M, Gobson S, Kimura S, Uchiyama Y, Polak JM, Masaki T, Kanazawa I. Endothelin: a novel peptide in the posterior pituitary system. *Science* 1990; **247**: 462–466.
6. Inoue H, Imokawa H, Yamanaka M, Isono E, Ando K, Kubota T, Aihara M, Ishida H. Detection of endothelin 1,2 and endothelin like immunoreactant in wound surface and plasma in mice with thermal injury. *Life Sci* 1993; **52**: PL291–PL296.
7. Boyce S, Ham RG. Calcium-regulated differentiation of normal human epidermal keratinocytes in chemically defined clonal culture and serum-free serial culture. *J Invest Dermatol* 1983; **81**: 33s–40s.
8. Kohno M, Horie T, Ikeda M, Yokokawa K, Fukui T, Yasunari K, Kurihara N, Takeda T. Angiotensin II stimulates endothelin-1 secretion in cultured rat mesangial cells. *Kidney Int* 1992; **42**: 860–866.
9. Shichiri M, Hirata Y, Emori T, Ohta K, Sato K, Sato A, Marumo F. Secretion of endothelin and related peptides from renal epithelial cell lines. *FEBS Lett* 1989; **253**: 203–206.
10. Bull HA, Bunker CB, Terenghi G, Springall DR, Zhao Y, Polak JM, Dowd PM. Endothelin-1 in human skin: immunolocalization, receptor binding, mRNA expression, and effect on cutaneous microvascular endothelial cells. *J Invest Dermatol* 1991; **97**: 618–623.
11. Yohn JJ, Morelli JG, Walchak SJ, Rundell KB, Norris DA, Zamora MR. Cultured human keratinocytes synthesis and secrete endothelin-1. *J Invest Dermatol* 1993; **100**: 23–26.
12. Yoshizumi M, Kurihara H, Morita T, Yamashita T, Oh-hashi Y, Sugiyama T, Takaku F, Yanagisawa M, Masaki T, Yazaki Y. Interleukin 1 increase the production of endothelin-1 by cultured endothelial cells. *Biochim Biophys Res Commun* 1990; **166**: 324–329.
13. Katabami T, Shimizu M, Okano K, Yano Y, Nemoto K, Ogura M, Tsukamoto T, Suzuki S, Ohira K, Yamada Y, Sekita N, Yoshida A, Someya K. Intracellular signal transduction for interleukin-1β-induced endothelin production in human umbilical vein endothelial cells. *Biochim Biophys Res Commun* 1992; **188**: 565–570.
14. Kurihara H, Yoshizumi M, Sugiyama T, Takaku F, Yanagisawa M, Masaki T, Hamaoki M, Kato H, Yazaki Y. Transforming growth factor-β stimulates the expression of endothelin mRNA by vascular endothelial cells. *Biochim Biophys Res Commun* 1989; **159**: 1435–1440.
15. Schmitt A, Hauser C, Jaunin F, Dayer JM, Saurat JH. Normal epidermis contains high amounts of natural tissue IL-1. Biochemical analysis by HPLC identifies and MW~ 17 Kd form with a PI 5.7 MW~ 30 Kd form. *Lymphokine Res* 1986; **5**: 105–118.
16. Kupper TS. Interleukin-1 and other human keratinocyte cytokines. *Adv Dermatol* 1988; **3**: 293–301.
17. Pentland AP, Mahoney MG. Keratinocytes prostaglandinsynthesis is enhanced by IL-1. *J Invest Dermatol* 1990; **94**: 43–46.
18. Yokokawa K, Mandal AK, Kohno M, Horie T, Murakawa K, Takeda T. Heparin suppresses endothelin-1 action and production spontaneously hypertensive rat. *Am J Physiol* 1992; **263**: R1035–R1041.
19. Yada Y, Higuchi K, Imokawa G. Effects of endothelins on signal-transduction and proliferation in human melanocytes. *J Biol Chem* 1991; **266**: 18352–18357.

**Received 4 May 1994;**
**accepted in revised form 4 August 1994**
